# Human iPSC-derived photoreceptor transplantation in the cone dominant 13-lined ground squirrel

**DOI:** 10.1016/j.stemcr.2024.01.005

**Published:** 2024-02-08

**Authors:** Ching Tzu Yu, Sangeetha Kandoi, Ramesh Periasamy, L. Vinod K. Reddy, Hannah M. Follett, Phyllis Summerfelt, Cassandra Martinez, Chloe Guillaume, Owen Bowie, Thomas B. Connor, Daniel M. Lipinski, Kenneth P. Allen, Dana K. Merriman, Joseph Carroll, Deepak A. Lamba

**Affiliations:** 1 Department of Cell Biology, Neurobiology, and Anatomy, Medical College of Wisconsin, Milwaukee, WI, USA; 2 Department of Ophthalmology, University of California, San Francisco, San Francisco, CA, USA; 3 Eli and Edythe Broad Center of Regeneration Medicine and Stem Cell Research, University of California, San Francisco, San Francisco, CA, USA; 4 Department of Ophthalmology and Visual Sciences, Medical College of Wisconsin, Milwaukee, WI, USA; 5 School of Medicine, Medical College of Wisconsin, Milwaukee, WI, USA; 6 Department of Microbiology and Immunology, Biomedical Resource Center, Medical College of Wisconsin, Milwaukee, WI, USA; 7 Department of Biology, University of Wisconsin-Oshkosh, Oshkosh, WI, USA

**Keywords:** 13-LGS, ground squirrels, photoreceptor transplantation, iPSC, retinal organoids, cone-dominant species, stem cells

## Abstract

Several retinal degenerations affect the human central retina, which is primarily comprised of cones and is essential for high acuity and color vision. Transplanting cone photoreceptors is a promising strategy to replace degenerated cones in this region. Although this approach has been investigated in a handful of animal models, commonly used rodent models lack a cone-rich region and larger models can be expensive and inaccessible, impeding the translation of therapies. Here, we transplanted dissociated GFP-expressing photoreceptors from retinal organoids differentiated from human induced pluripotent stem cells into the subretinal space of damaged and undamaged cone-dominant 13-lined ground squirrel eyes. Transplanted cell survival was documented via noninvasive high-resolution imaging and immunohistochemistry to confirm the presence of human donor photoreceptors for up to 4 months posttransplantation. These results demonstrate the utility of a cone-dominant rodent model for advancing the clinical translation of cell replacement therapies.

## Highlights

• Successful integration of hiPSC photoreceptors was observed in the 13-LGS retina
• Noninvasive longitudinal imaging traced transplanted cells for up to 4 months
• Maturation structures resembling inner segments was visualized using AOSLO imaging
• Degree of integration was related to type of damage in the induced 13-LGS models

## Teaser

Yu and colleagues showed that human iPSC-derived photoreceptors generated from 3D-retinal organoids could survive up to 4 months following transplantation in the cone-dominant 13-lined ground squirrel retina. The degree of integration and intraretinal localization of the transplanted cells appeared to depend on the degree of retinal damage.

## Introduction

The evolution of stem cell and retinal organoid technologies has generated remarkable opportunities for therapeutic development, including precision medicine and cell replacement therapies. *In vitro*–derived three-dimensional (3D)-retinal organoids are “mini-retinas” that faithfully mimic the *in vivo* retinogenesis timeline to make each of the seven major cell types of retinal tissues sequentially in a self-assembled fashion. These retinal organoids are used as a mainstream tool for advancing numerous studies, including organoid development, disease modeling, drug discovery, gene therapy, regenerative medicine, and precision medicine (Kandoi and Lamba, 2023). Stem cell–derived photoreceptors have been shown to successfully survive and integrate into host retinas of numerous animal models following transplantation, including mice and rats (Lamba et al., 2009, 2010; Mandai et al., 2017; McLelland et al., 2018; Zhu et al., 2017) and, more recently, in canines (Ripolles-Garcia et al., 2020). However, all of the models used to date have experimental limitations. For example, traditional rodent models lack a cone-rich central region resembling the human macula, which makes it difficult to test therapies for many macular or cone dystrophies. Although larger animal models such as canine, swine, and nonhuman primates (NHPs) have a retinal architecture that mimics the cellular landscape of the human macula (Aboualizadeh et al., 2020; Chandler et al., 1999; Mowat et al., 2008), these models can be expensive, have limited availability, and lack the expansive transgenic tools that are available in rodents.

Despite these limitations, the ability to use noninvasive imaging to monitor cell survival and integration increases the utility of all animal models. For examples, scanning light ophthalmoscopy (SLO) and optical coherence tomography (OCT) have been used in several studies to monitor transplanted cells *in vivo* in mice and NHPs (Liu et al., 2020; Takagi et al., 2018; Uyama et al., 2022). While the noninvasive nature of these imaging approaches enables longitudinal study, they have limited lateral resolution. Adaptive optics techniques have recently been applied to numerous animal models (Geng et al., 2012; Huckenpahler et al., 2019; Hunter et al., 2010; Joseph et al., 2020; Sajdak et al., 2016), which enable cellular resolution imaging of retinal cells. Noninvasive fluorescence adaptive optics SLO (AOSLO) has also been used to track the survival and migration of individual transplanted photoreceptors in cynomolgus monkey eyes (Aboualizadeh et al., 2020). Coupling these advances in imaging with novel animal models offers an opportunity to move retinal therapeutics forward.

Here, we sought to advance cone replacement therapy efforts by exploring nonconfocal AOSLO imaging in the 13-lined ground squirrel (13-LGS). Advantages of the 13-LGS include the unique “cone-rich” retina (Ahnelt, 1985; Jacobs and Yolton, 1969), low cost and ease of availability, and amenability to noninvasive retinal imaging. In the present study, we have assessed the survival and integration of fluorescently labeled photoreceptors derived from human retinal organoids in 13-LGS with healthy, undamaged control retinas as well as mechanically or chemically damaged retinas. Retinal damage was induced with chemical intravitreal injection or mechanically by retinal detachment in the inferior retina. Following transplantation, transplanted cells were longitudinally tracked to examine survival. Using nonconfocal AOSLO, we were able to resolve structures resembling cone inner segments in the transplanted region with concomitant fluorescent signal captured through SLO. The *in vivo* data were corroborated with immunohistochemical studies that demonstrated the presence of human and retinal cell-type-specific markers for up to 4 months posttransplantation in these retinas. Our results demonstrate the utility of this alternative cone-rich preclinical model for advancing cone replacement therapeutic approaches.

## Results

### Long-term survival of human induced pluripotent stem cell (hiPSC)–derived retinal cells up to 4 months posttransplantation

An hiPSC line with GFP knocked in at the *AAVS1* safe harbor locus was differentiated into cone-rich 3D-retinal organoids (Figures S1A–S1C) (Pei et al., 2015). The 60-day-old organoids were briefly pulsed (24 h) with a Notch inhibitor (PF-03084014) to drive the fate into cone differentiation and reduce the proliferative stem cell population, as previously described (Chew et al., 2022). 13-LGS retinas were damaged at least 2 weeks pretransplantation through either chemical insult or mechanical detachment (Figures S2A–S2C). The horizontal optic nerve head (ONH) in 13-LGS eyes was used as a landmark to distinguish the degenerated inferior versus nondegenerated superior retinal region. Upon the receipt of organoids from the University of California, San Francisco (UCSF) at the Medical College of Wisconsin (MCW), the organoids were dissociated into single cells and their viability was assessed to be 61.2%–93.5% by flow cytometry. The average viability obtained was 77.18% ± 8.08% from a total of 11 shipments of organoids for transplantation conducted over a period of 3 years (Figures S1D and S1E).

**Figure 1:**
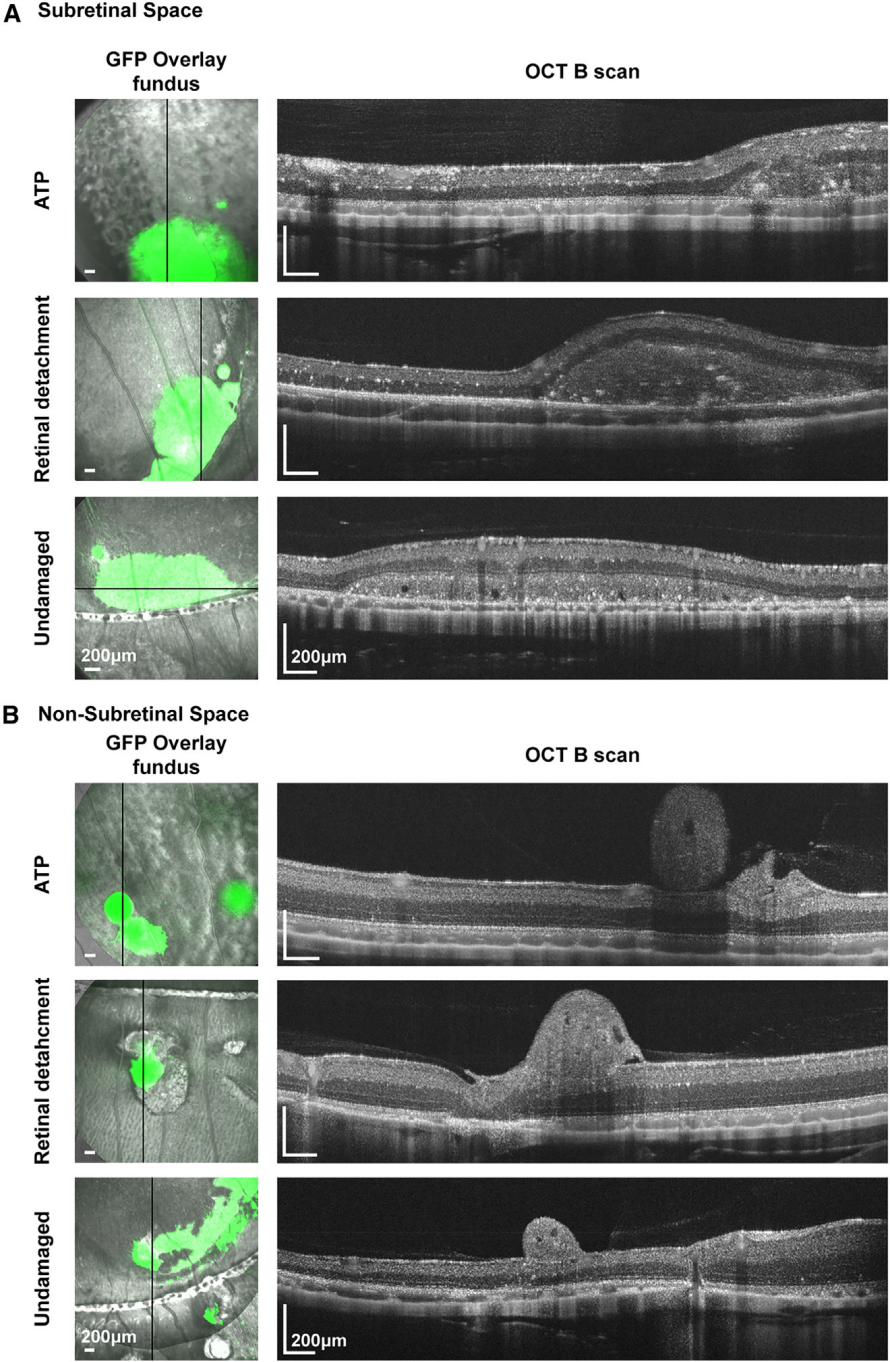
Localization of transplanted cell in ATP, retinal detached, and undamaged 13-LGS retina (A and B) (A) Representative fundus images of fSLO overlaid on NIR images and their corresponding OCT B scan images in damaged (ATP, retinal detachment) and undamaged 13-LGS retinas showing the localization of transplanted cells in the SRS and (B) in the non-SRS, including vitreous and epiretinal surface. The black line in the fundus images of (A) and (B) corresponds to the cross-sectional location of the SD-OCT B scan. Scale bar, 200μm.

Approximately 0.7–1 million cells dissociated from the retinal organoids were injected into the subretinal space (SRS) of 13-LGS eyes. The animals were immunosuppressed by adding 210 mg/L of cyclosporine A (AC457970050, Thermo Fisher Scientific, Waltham, MA) in their drinking water for 2 weeks (1 week before and 1 week after transplantation) to prevent perioperative cell rejection. Posttransplantation, the presence and the location of the transplanted bolus of cells was visualized immediately by fluorescent SLO (fSLO) and spectral domain OCT and compared against the pretransplantation baseline condition images. Longitudinal retinal imaging collected monthly indicated the presence of GFP signal from transplanted cells up to 16 weeks in both ATP-induced and retinal detachment–damaged models (Figure S2D). The presence of fluorescence signal persisted in 11/16 eyes of ATP-damaged and in 11/18 eyes of retinal detachment models, for up to 16 weeks. In the undamaged controls, 8/20 eyes showed the survival of transplanted cells up to 12 weeks. Although we primarily delivered the cells into the SRS, reflux of injected cells was observed in the non-SRS space along the needle track in some of the transplanted retinas (Figure 1). These transplanted cells settled in different retinal layers within the 13-LGS retinas (Figure 1A) or in the vitreous space/epiretinal location (Figure 1B). Overall, monthly follow-up using fSLO fundus and OCT *in vivo* imaging confirmed the presence of cell clumps in the non-SRS (vitreous space, epiretinal location) and SRS of degenerated and undamaged control models (Figure 1).

### Donor photoreceptors’ inner segment structure, colocalized with the persistent GFP patch at 3 months postinjection

**Figure 2:**
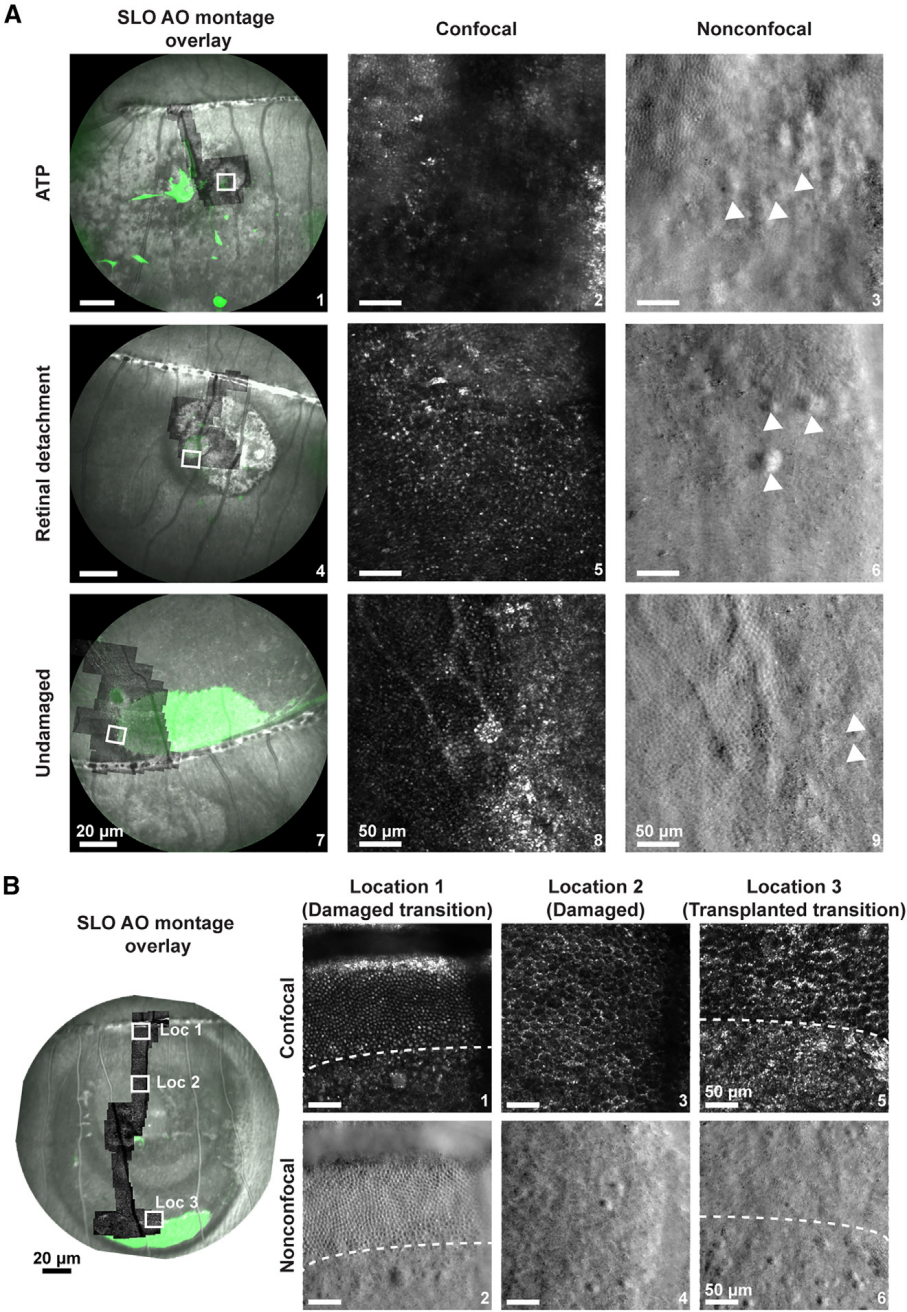
Adaptive optics (confocal and nonconfocal) images of damaged and undamaged 13-LGS retinas (A) Representative fSLO images overlaid on AOSLO montages from ATP, retinal detached, and undamaged 13-LGS retinas exhibiting the localization of the transplanted region (A-1, A-4, and A-7). AOSLO confocal (A-2, A-5, and A-8) and nonconfocal (A-3, A-6, and A-9) magnified images of the white boxed region on the transplanted ROI (A-1, A-4, and A-7) showing the reflective and scattering pattern of the photoreceptor outer segments and inner segments from a single photoreceptor cell, respectively. ATP and retinal detachment models show a complete loss of outer segments (A-2 and A-5). The transplanted cells in all 3 models show putative inner segments (white arrowheads, A-3, A-6, and A-9). (B) A representative fSLO image overlaid on AOSLO montage from the retinal detachment model. The confocal and nonconfocal images captured using AOSLO at the damaged transition border (B-1 and B-2), within the damaged region (B-3 and B-4), and at the transplanted transition border (B-5 and B-6) is shown here. The dotted white line marks the boundary separating the nondetached and detached retinal regions (B-1 and B-2). The image shows the intact outer segment and inner segments above the white dashed line area in the undamaged retinal region versus the loss of the cone mosaic below the white dashed line area in the detached retinal region (B-1 and B-2). Complete degeneration of cone mosaic with hexagonal packaging of the underlying RPE is shown in the damaged images (B-3 and B-4). The confocal and nonconfocal images in the transplanted transition region show reflective patterns instead of outer segment structure and putative inner segments in the transplanted region (below the white dashed line), respectively (B-5 and B-6). Scale bar, 50μm.

**Figure 3:**
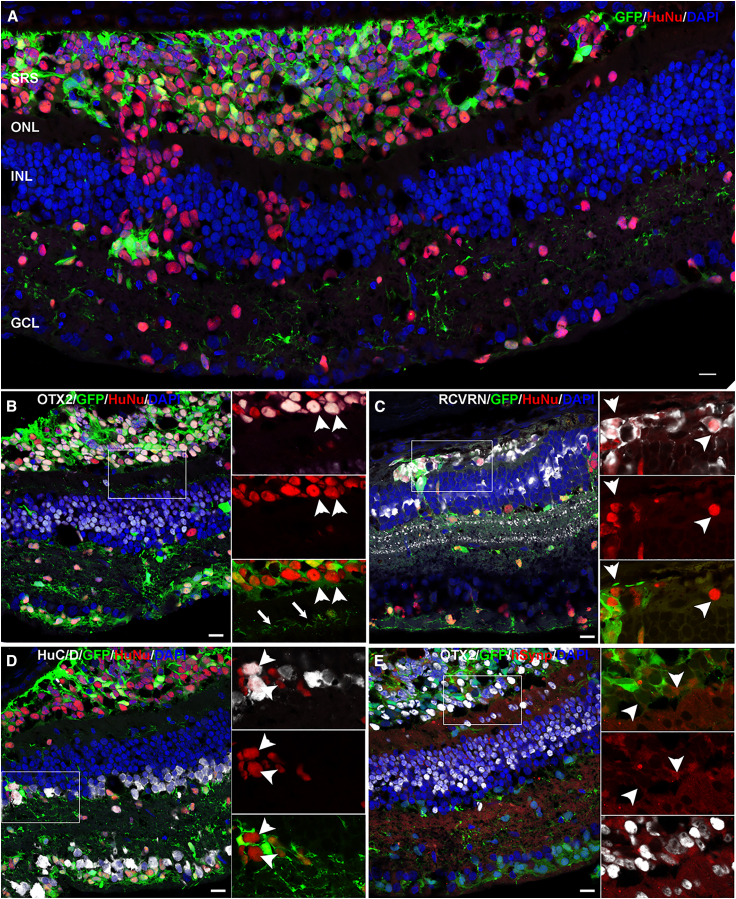
*Ex vivo* analysis of transplanted cells in ATP-induced damaged retina (A) Representative images of the transplanted regions showing colocalization of surviving and integrated GFP^+^ cells with the human nuclear marker HuNu (red). The human marker–positive cells were observed to be scattered across the various retinal layers. (B and C) Cells in the outer retina colabeled with the photoreceptor markers OTX2 (B, white) and recoverin (C, white). (D) Representative image showing HuNu^+^ (red) and GFP^+^ cells in the inner retinal layers colocalizing with the amacrine/ganglion cell marker, HuC/D (white) (B). (E) Representative image showing expression of synaptic marker synaptophysin (red) using a human-specific antibody in GFP^+^ cells in the outer retina. Insets for each subpanel show a magnified view and arrowheads highlighting the coexpression. The arrows in (B) highlight GFP^+^ processes extending into the plexiform layer. DAPI (blue) marks nuclei. INL, inner nuclear layer, GCL, ganglion cell layer. Scale bar, 20 μm.

**Figure 4:**
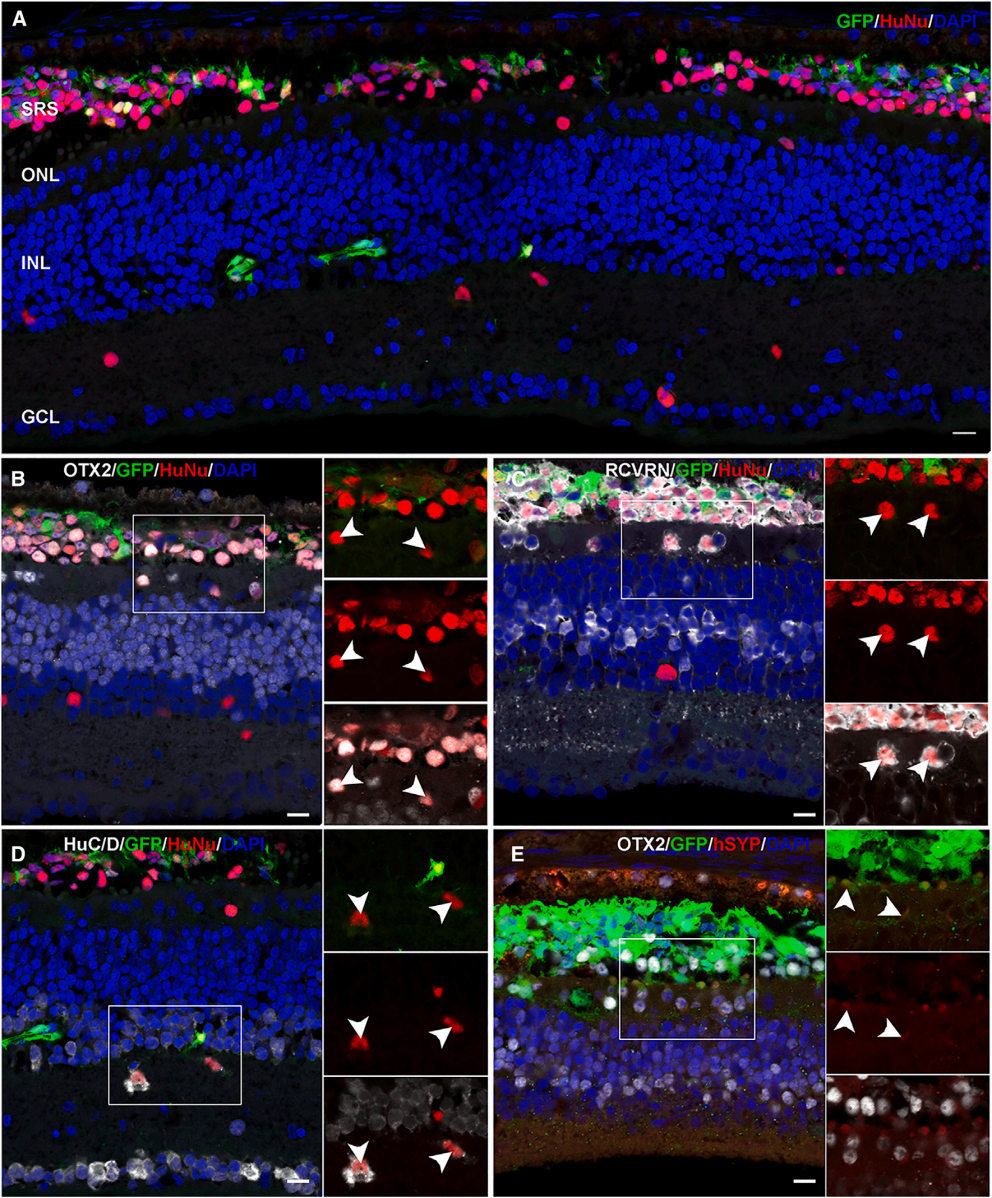
*Ex vivo* analysis of transplanted cells in retinal detachment–damaged retina (A) Representative images of the transplanted regions showing colocalization of surviving and integrated GFP^+^ cells with the human nuclear marker HuNu (red). The human marker–positive cells were observed to be scattered across the various retinal layers. (B and C) Cells in the outer retina colabeled with the photoreceptor markers OTX2 (B, white) and recoverin (C, white). (D) Representative image showing HuNu^+^ (red) and GFP^+^ cells in the inner retinal layers colocalizing with the amacrine/ganglion cell marker HuC/D (white) (B). (E) Representative image showing expression of synaptic marker synaptophysin (red) using a human-specific antibody in GFP^+^ cells in the outer retina. Insets for each subpanel show a magnified view and arrowheads highlight the co-expression. Scale bar, 20 μm.

At the end stage of either a 6-week or 12-week follow-up, the retina was imaged with a custom AOSLO system to visualize the transplanted cells with single-cell resolution. We confirmed the presence and identified the location of the transplanted region through fSLO imaging. Then, we further sorted the transplanted retina by the location of cell clumps in different retinal layers via SD-OCT imaging (Figure 1). Through the fSLO and AOSLO montage overlay, we selectively targeted the region where the cells resided in each model; ATP, retinal detachment, and undamaged eyes (Figure 2A). We further extracted one region of interest (ROI) from the AOSLO montage, and the results were presented in confocal and nonconfocal modalities (Figure 2A). The confocal modality collected the light reflected from the photoreceptor outer segments, whereas the nonconfocal modality collected light that was scattered from the photoreceptor inner segments (Scoles et al., 2014). Evidence of host photoreceptor degeneration was noted in ATP and retinal detachment models via AOSLO by disruption of the host cone photoreceptor mosaic (Figures 2A-2 and 2A-5). Furthermore, the region of the transplanted cells captured on the confocal channel was observed as a reflective material at 150 days postinjection. This result was not unexpected because the photoreceptor outer segments do not develop until 180–200 days in retinal organoid cultures for the hiPSC line used in this study. Further tracking of these specific cells via nonconfocal imaging indicated the presence of structures resembling photoreceptor inner segments in the transplanted region in the retinal detachment model (Figure 2B). These mature photoreceptors were not visualized within the ATP model. This result suggests that the transplanted cells in the ATP model animals were not uniformly bounded in the photoreceptor layer based on *in vivo* imaging.

Further comprehensive image analysis on the retinal detachment model was performed where AOSLO montages were spatially aligned to the fSLO image to colocalize the GFP fluorescent signal (Figure 2B). Visualization of the transplanted cells was seen most inferiorly within the subretinal detachment, likely due to gravitational effects (Figure 2B). The three locations presented are the transition/border of the subretinal detachment, within the detachment, and at the transition zone to the injected cells (Figure 2B). The morphology of the unaffected cone mosaic with intact inner and outer segments is shown above the white dashed lines in the damaged transition images acquired by confocal/nonconfocal imaging (Figures 2B-1 and 2B-2). In contrast, the areas below the white dashed lines primarily show the disrupted cone mosaic and some enlarged photoreceptors, which is likely due to edematous swelling, that occurs secondarily to damage (Figures 2B-1 and 2B-2). A complete loss of photoreceptors in the ablated region was seen, as evidenced by the exposed hexagonal packing pattern of the underlying the retinal pigmented epithelial cells (Figures 2B-3 and 2B-4). Lastly, examination of the confocal images in the transplanted transition zone of injected cells revealed a reflective tissue covering the ablated region below the white dashed line compared to the nontransplanted/damaged region seen above the white dashed line (Figure 2B-5). Although resemblance of a photoreceptor mosaic was not seen, our imaging demonstrates the presence of injected material in the region below the white dashed lines in transplanted transition images (Figure 2B-5). In the nonconfocal modality image of the transplanted transition region, the presence of potential replacement photoreceptors is demonstrated by structures resembling the inner segments of the photoreceptors in the region below the white dashed line (Figure 2B-6). To sum up, the *in vivo* imaging data show both the survival and maturation of donor photoreceptors following the subretinal transplantation in the 13-LGS retinal detachment model up to 16 weeks.

### Transplanted donor cell was identified as either photoreceptor/ganglion/amacrine cell by histology

Following postmortem tissue collection, retinal sections were stained with the previously described human nuclear marker (HuNu) (Zhu et al., 2017) and cells expressing GFP were confirmed to colocalize it (Figures 3A and 4A). Furthermore, we identified more human nuclear marker–expressing cells compared to GFP, likely due to the silencing of GFP promoters as cells mature. We confirmed this by assessing GFP expression in D150 organoids from this line. We observed lower or lack of GFP signal in OTX2^+^ photoreceptors and especially HuC/D^+^ (ELAVL3/ELAVL4) inner retinal neurons while it was robustly present in VSX2^+^ retinal progenitors (Figures S1F and S1G). We further confirmed human origin with another human-specific marker, human LMNB2 (Figure S4A). Interestingly, we also observed that the photoreceptor loss in the host retina was complete in the ATP model relative to the retinal detachment model when comparing the DAPI channel in the outer nuclear layer (ONL) (Figures 3A, 4A, and S2B). The retinal detachment model still had a partially preserved photoreceptor layer (Figure 4A and S2C). Next, we explored the fate of the HuNu^+^ cells. Cells transplanted in ATP-induced damaged retinas were found to be scattered axially across the retina (Figure 3). This suggests that the cells were able to migrate across the outer limiting membrane and integrate into different retinal layers. In contrast, the transplanted cell in the retinal detachment model were mainly confined within the subretinal space, with very few cells migrating into the retina (Figure 4). Based on these observations, we conclude that the migration of transplanted photoreceptor cells into the retina can occur but depends on the severity of damage.

We next evaluated the fate of the transplanted cells. Overall, the majority of the transplanted cells were found to be OTX2^+^ photoreceptor with some inner retina migrating cells expressing amacrine/ganglion marker (HuC/D^+^) in both ATP and retinal detachment models (Figures 3B, 3C, 4B, and 4C). The HuNu^+^ cells in the subretinal space of both ATP and retinal detachment models and a few that migrated into surviving host ONL were identified as photoreceptors by co-staining with the photoreceptor marker Otx2 (Figures 3B and 4B), with GFP^+^ processes extending into the plexiform layer, where photoreceptor synapse with bipolar and horizontal cells. The majority of the HuNu^+^/OTX2^+^ cells also co-stained for another photoreceptor transduction marker, recoverin (Figures 3C and 4C). Finally, we confirmed the presence of human cones in some of the transplanted GFP^+^ cells by the expression of retinoid X receptor gamma, cone arrestin (ARR3), and green cone opsin (GCO), although the expression of the mature markers (GCO and ARR3) was much lower than that in host photoreceptors (Figure S3). The HuNu^+^ cells that migrated into the inner nuclear and ganglion cell layers were HuC/D^+^, suggestive of ganglion and amacrine cell fate (Figures 3D and 4D). Further analysis with the ganglion cell-specific marker BRN3A (POU4F1) showed that these cells were BRN3A^−^, suggestive of amacrine fate (Figure S4B). We also observed some migration of SOX2^+^/HuNu^+^ cells, indicating the limited migration ability of either stem cells or glia (Figure S4C). Finally, we assessed synaptic development by staining for a human-specific synaptic marker synaptophysin (Ludwig et al., 2023). We observed coexpression of the synaptic marker in GFP^+^ cells in both sets of transplant conditions (Figures 3E and 4E). Overall, the localization of the transplanted cells varied depending on the damage modality and the fate varied depending on the destination of the cell within the retina.

## Discussion

This study explored the survival and integration of transplanted hiPSC-derived photoreceptors in the cone-dominant 13-LGS retina. Here, we emphasize our focus on the survival of the photoreceptor precursors in the cone-dominant environment and compared different damage models. For this study, we adopted a xenotransplantation approach to successfully demonstrate the survival of 60-day-old photoreceptors (from 3D-retinal organoids) in both chemically (ATP) and mechanically retinal detachment–induced retinal degeneration models of 13-LGS for up to 16 weeks posttransplantation. *In vivo* imaging modalities were used to consistently track the presence of GFP signal expressed by the transplanted human donor cells in the host retina. Furthermore, we imaged the transplanted location with a custom AOSLO system to evaluate the structure of these cell clusters compared to undamaged retinas with high-resolution imaging. Although this is not direct proof that these are indeed developing inner segments from the donor photoreceptors, it is encouraging because it resembles the structure of the photoreceptor inner segments. In a prior transplantation study within NHPs, fluorescence AOSLO was used to directly identify transplanted donor cells (Aboualizadeh et al., 2020).

Use of the 13-LGS model provides an advantage over previously reported transplantation studies using traditional rodent (e.g., mouse, rat) models due to their unique cone-dominant, rather than rod-dominant, retinal composition. This difference offers a host retina with an environment potentially better suited to recapitulate the human macula, which is cone dominant and drives some of our most important visual functions. Although retinal transplantation has been tested in NHPs (Shirai et al., 2016), which have a fovea like the human retina, our 13-LGS model is relatively less expensive and more easily accessible (Merriman et al., 2012). The main limitation of the 13-LGS is its natural cycle of hibernation from October to March (Sajdak et al., 2019). During hibernation, the 13-LGS retina undergoes extensive retinal remodeling (Sajdak et al., 2019), which can confound long-term studies because the impact of remodeling on the transplanted cells is unknown. This may limit carrying out longer-term functional studies as retinal organoid derived human photoreceptors completely mature and express opsins at >180–200 days (Capowski et al., 2019; Kandoi and Lamba, 2023).

In transplantation studies, *ex vivo* histology results have the advantage of presenting direct evidence of the survival and integration of donor cells. While this provides information for a specific time point of assessment, the progression of cell survival and integration over time is lost. Conversely, our study adopted multimodal noninvasive imaging of the transplanted cells. This allowed for the longitudinal tracking of the survival of individual cells and integration over a 16-week period. The real-time tracking of viable cells is beneficial because it allows us to both continuously monitor the presence of donor cells, their position, and integration time point into the host retina. In addition, the noninvasive imaging methods SLO and SD-OCT, used to capture fluorescence signal and cell location here, are also commonly used clinical tools, highlighting the clinical translational capabilities of results obtained by these methods to future human clinical trials. Although noninvasive imaging offers the ability to continually capture information of cell survival and integration progression, it remains insufficient as direct evidence for characterizing retinal changes. Hence, the *in vivo* phenotypic findings would ultimately need confirmatory histology data to support our outcomes. Here, we successfully combined the dual approach of *in vivo* imaging along with histology at 16 weeks posttransplantation to confirm the integration status and the donor cell origin. Follow-up studies may be considered to validate the phenotypic findings of *in vivo* imaging corresponding to *ex vivo* evidence at each of the defined time points.

Several previous rodent studies suggest that the donor cells can transfer protein into the host cells and confound transplant data analysis (Pearson et al., 2016; Santos-Ferreira et al., 2016). This is thought to be predominantly due to cytoplasmic exchange through nanotube connections (Heisterkamp et al., 2022; Ortin-Martinez et al., 2021). However, in our findings, the material transfer of cytoplasmic protein is unlikely to confound our integration data, because GFP^+^ cells expressed at least two different human-specific markers. Interestingly, although most of the transplanted cells were OTX2-expressing photoreceptors, some human cells that migrated further into the ganglion cell layer or inner retina expressed the amacrine cell marker. This demonstrates the migration potential of these cells into the retina from the subretinal space where they were originally transplanted (Figures 3D and 4D). However, the degree of migration seen was dependent on the degree of photoreceptor loss in the damage model used. A complete disruption of the photoreceptor layer and possibly the outer limiting membrane seems to be required for large-scale migration into the inner retina. Another study in a murine model showed the incorporation of hiPSC-derived cones in a retina with cone degeneration (Gasparini et al., 2022). Future studies with more detailed characterization of the microenvironmental factors contributing to migration and integration of transplanted hiPSC-derived cones in such transplantation studies can provide clues for successful therapies.

## Experimental procedures

### Resource availability

#### Lead contact

Further information and requests for resources and reagents should be directed to and will be fulfilled by the corresponding author, Deepak A. Lamba (deepak.lamba@ucsf.edu).

#### Materials availability

This study did not generate new unique reagents.

#### Data and code availability

This study did not generate new datasets.

### Animals

The 13-LGS (*Ictidomys tridecemlineatus)* used in this study were obtained from the colony maintained at the University of Wisconsin-Oshkosh (Merriman et al., 2012; Vaughan et al., 2006). All of the experimental procedures were described and approved by the Institutional Animal Care and Use Committee at the MCW (protocol no. AUA00005654). The experiments of this study were carried out over 3 consecutive years during the active nonhibernating season (April–October 2020–2022). A total of 88 eyes were injected with hiPSC-derived photoreceptors from 3D-retinal organoids in 11 batches. This included control wild-type eyes (n = 10), ATP-induced damaged eyes (n = 16), and retinal detachment eyes (n = 62). For the retinal detachment eyes, only the inferior retina was detached. In addition, in the retinal detachment group, 20 eyes were used as experimental undamaged controls by injecting them in the superior region of the retina. The injection of cells in the undamaged retina was done as a sentinel to determine the success of the xenotransplantation.

### Anesthesia and eye preparation for transplantation and imaging

All of the 13-LGS were anesthetized using isoflurane mixed with oxygen. The animals were first induced with a 5% isoflurane (901805, VetEquip, Livermore, CA) inhalant in a clear induction chamber. The isoflurane was maintained at 5% throughout the surgical procedures through a nose cone. During the imaging procedures, the isoflurane was maintained at 1%–4% through a nose cone. Isoflurane exposure to investigative staff was minimized by passive gas scavenging. Following isoflurane induction, the eyes of the 13-LGS were dilated and cyclopleged with 2.5% phenylephrine (17478020102, Akron, Lake Forest, IL) and 1% tropicamide (17478010212, Akron). Next, the animal was positioned in a preheated platform for transplantation. For imaging, the animal was positioned in an imaging cassette with a custom mask mount to hold the nose cone in place. Each imaging cassette had a nodal point in which the eye was aligned to minimize the adjustment of the position of the animal after the initial setup.

### Retinal injections in 13-LGS

Retinal degenerations were induced using chemicals via intravitreal injection of ATP (AAJ1058509, Thermo Fisher Scientific, Waltham, MA) or mechanically by subretinal injection of 0.9% normal saline (012007, Phoenix, Manhattan, KS). Before all of the retinal surgical procedures, the dilated and cyclopleged eyes received a drop of tetracaine (10UEF, Alcon, Fort Worth, TX) to provide a temporary local anesthetic. The eye was dropped with betadine (405943, Alcon) to sterilize the region surrounding the injection site and minimize the chances of infection from injections. Next, the top of the eye was coated with Gonak solution (9050-1, Sigma Pharmaceuticals, North Liberty, IA) and a small sterile circular coverslip was used to facilitate the visualization of the fundus. Next, 10-μL intravitreal injections of 0.723 M ATP were injected with a 1-cm^3^ insulin syringe closer to the retinal surface in the vitreous space. For retinal detachment, the needle was first placed into the subretinal layer and 50–75 μL of 0.9% sterile normal saline was injected into the inferior retina below the ONH. Degenerations were induced at least 2 weeks before cell transplantation. Cells were transplanted via subretinal injection by using a 25G trocar to make a small 1-mm insert from the limbus, and ∼0.7–1 million dissociated cells (in 40 μL of 3D-retinal differentiation medium [RDM] with all-*trans* retinoic acid [ATRA]) from the retinal organoids were injected into the degenerated inferior retina and experimental undamaged superior retina using a Hamilton syringe fitted with a 33G blunt needle. A Leica microsystem M651 surgical scope (Leica Biosystems, Wetzlar, Germany) or a Leica intraoperative surgical scope Proveo8 (Leica Biosystems) was used to visualize the retina during injections.

### Human retinal organoid differentiation

All human stem cell studies were approved by UCSF Institutional Review Board (IRB) and Human Gamete, Embryo and Stem Cell Research (GESCR) Committees. hiPSCs knocked in with pan-expressing CAG-GFP at the safe harbor locus (Pei et al., 2015) were directed toward retinal fate via the embryoid body (EB) and three-step (3D-2D-3D) differentiation approach, as per the published protocols from our previous studies (Arthur et al., 2022; Bachu et al., 2022). Briefly, hiPSCs were lifted and cultured onto a suspension plate to form EBs. After 1 week, the EBs were briefly exposed to BMP4 at varying concentrations (1.5–0.375 nM) for approximately 1 week. The EBs were then plated onto a Matrigel-coated plate to form optic vesicles in RDM. At the end of 4 weeks, the optic vesicles were manually excised and cultured in a 3D-RDM with 1 μM ATRA (R2625, Sigma-Aldrich, St. Louis, MO) to generate self-assembled laminated 3D-retinal organoids. Approximately 55- to 60-day-old organoids were treated with 10 μM PF-03084014 hydrobromide (PF) (PZ0298, Sigma-Aldrich), a small-molecule inhibitor of the Notch pathway for 24 h. The purpose of using PF was to drive the differentiation of the remaining retinal progenitor pool within the 3D-retinal organoids toward the cone fate (Chew et al., 2022). PF media was removed and viable cone-rich 3D-organoids expressing the parental reporter GFP were then collected in a 15-mL conical tube filled with freshly prepared 3D-RDM containing ATRA. The tube with PF-treated organoids was then shipped from UCSF to MCW overnight with warm packs (by FedEx overnight priority delivery) for next-day cell transplantation.

### Organoid dissociation and cell viability assay

On the transplant day, the retinal organoids were briefly washed with 1× PBS, centrifuged at 50 × *g*, and treated with 5 mL papain solution containing Earle’s Balanced Salt Solution and 500 μL deoxyribonuclease I (LK003150, Worthington Biochemical, Lakewood, NJ) for 30–45 min with gentle agitation and a couple of intermittent triturations until complete dissociation. Postdissociation, the cells were passed through a 100-μm cell strainer (22-363-549, Fisher Scientific, Pittsburgh, PA). The cells were quantified using 0.4% trypan blue dye (1525006, Thermo Fisher, Waltham, MA) on a hemocytometer. Lastly, the cells were pelleted at 200 × *g* and were resuspended in the 3D-RDM with ATRA at the desired cell concentration (2.8 × 10^4^–3.5 × 10^4^ cells/μL) for injection.

### Cell viability assay using flow cytometry

Dissociated cells 1 × 10^6^ were fixed with 2% paraformaldehyde (28908, Thermo Scientific, Waltham, MA) for 5 min and washed with 1× PBS by centrifugation at 200 × *g* for 5 min. Cells were then incubated in the dark with 1 μM TO-PRO-3 staining solution (T3605, Thermo Fisher) in 1 mL 1× PBS for 15 min. Following fixation and staining, cells were washed 3 times with 1× PBS by centrifugation at 1,200 rpm for 5 min. The final cell pellet was resuspended in flow cytometry buffer (Ca/Mg^2+^-Free PBS, 10010023, Thermo Fisher + 2% fetal bovine serum, 16000044, Thermo Fisher + 0.1% sodium azide, 71448-16, Fisher Scientific) for analysis. The unstained fixed cells were used as control for optimizing the gating strategy. Acquisition of the cells was performed using a BD LSR-II with appropriately set parameters (642 nm excitation/661 nm emission) and analyzed using flow cytometry analysis software (FlowJo version 10.7.2). Live cells were quantified after excluding the dead cell population stained by TO-PRO-3.

### Retinal imaging

#### SLO

GFP signals from the transplanted cells in the host retina of 13-LGS were assessed by *in vivo* fundus imaging. The Spectralis HRA with a customized multiline system (Heidelberg Engineering, Heidelberg, Germany) was used to capture the images. The near-infrared reflectance (NIR) images were captured using an 810- to 820-nm laser source. The GFP images were captured using an excitation laser (peak = 486 nm, full width at half-maximum = 4 nm) and transmission filters (500–550 nm). The NIR and the GFP images were captured by registering and averaging 30–50 and 100 frames, respectively. The sensitivity of images was set at 36%–40% and 100% for NIR and GFP, respectively. The animals were anesthetized as described earlier for the imaging session. The images were then captured starting at the ONH and then moving toward the inferior/superior retina for visualizing the GFP signals at the bleb location.

#### SD-OCT

Cross-sectional *in vivo* retinal images were captured using Bioptigen Envisu R2200 or R2310 SD-OCT systems equipped with a rabbit imaging bore (Leica Microsystems). The animals were anesthetized as described earlier for the imaging session. The baseline imaging on the degenerated retinas at pretransplantation (1 week) and posttransplantation (2, 6, 12, and 16 weeks) were captured for each animal. The R2200 and R2310 SD-OCT systems were used interchangeably, and the image was registered to the same scale while processing. For each imaging session, vertical and horizontal volume scans were acquired using 650 A scans/B scans and 300 B scans per volume parameters. In addition, vertical and horizontal line scans (1,000 A scans/B scans with 100 repeats of B scans) were acquired following each volume scan. Volume scans were used to locate the ROIs within the retina, and line scans were used to capture repeated scans at the exact location of interest. The images were taken along the ONH for baseline imaging, and the horizontal ONH was used as a landmark for aligning baseline and follow-up images. Once the image was acquired, the volume scans were processed and segmented using in-house customized processing software, OCT volume viewer, to extract the precise fundus image with the location of the line scan. The line scans were then processed using ImageJ (NIH, Bethesda, MD) software. Among the 100 frames, 25 frames with the least movement were extracted, and 1 reference frame was extracted from the 20 frames. The reference frame was used to align the 25 frames, and the selected 20 frames were averaged as the final processed image.

#### AOSLO

A high-resolution image of the cone mosaic of transplanted cells was captured using our previously described custom AOSLO system (Gaffney et al., 2021; Sajdak et al. 2016, 2019). The animal was anesthetized and dilated as described earlier. The transplanted regions within the retinas were imaged 12 weeks before endpoint euthanasia. The fundus image collected with fSLO provided a road map for the AOSLO imaging (Figures 2A-1, 2A-4, and 2A-7). The protocol collected the outer retinal layer structure starting at the ONH and across the subretinal detachment region to the transplanted cells. Confocal and nonconfocal modality images were captured simultaneously to detect the outer and inner segments of the photoreceptor. During each imaging session, a segment of the optic nerve or blood vessel was captured as a landmark. In addition, a montage of the transplanted location was captured. The lid of the eye was held open with a speculum as needed, and lubrication drops were applied with either artificial tears (TRS-05-GCP, Gericare, Brooklyn, NY) or Systane (00065143105, Alcon).

The custom AOSLO system used a 790-nm superluminescent diode (SLD) laser source for imaging and an 850-nm SLD for wavefront sensing. The measured optical power of 790 nm and 850 nm was 355 μW and 48 μW, respectively. Eye wavefront aberration was measured with a Shark-Hartmann wavefront sensor and corrected with a 7.2-mm diameter 97-actuator ALPAO deformable mirror (ALPAO, Montbonnot-Saint-Martin, France). The system was modified to image an animal eye with a 4.5-mm system pupil diameter. The raw data were collected as 100-frame video image sequences. The image was captured with 2° or 3° fields of view. The custom image registration and processing steps were completed as previously described (Sajdak et al., 2016, 2019). The collected image sequences were first desinusoided to remove the distortion due to the sinusoidal motion of the resonant scanner with a Ronchi ruling grid of 118.1 lines/mm image to estimate the distortion. The collected image sequences were next examined, and a reference frame was selected for registration. With the selected reference frame, the image sequences were strip registered through the custom software to display the processed image. Finally, the processed images were semiautomatically montaged and aligned by a custom MATLAB script with a locations file for each image. The aligned images were imported in Adobe Photoshop (Adobe, San Jose, CA), and the alignment of each image was manually checked and adjusted to create a final montage.

### 13-LGS eye collection and retinal section preparations

The animals were humanely euthanized by decapitation following isoflurane anesthesia. The whole eye globe with a GFP signal collected from the last *in vivo* imaging time point was extracted from the animals. The extracted eye was initially rinsed with fresh cold 1× PBS, and a slight opening was created at the limbus of the eye with a small blade. The whole eye was next immersed in freshly prepared cold 4% paraformaldehyde (157-8, Electron Microscopy Sciences, Hatfield, PA) prepared in 0.1 M sodium phosphate buffer. The fixed eye was left at 4°C overnight and was washed with cold 0.1 M sodium phosphate buffer 3 times with 15-min intervals. Then, the anterior segment, the lens, and the vitreous humor were dissected from the eye globe. The fixed eye cups were immersed again in cold 4% paraformaldehyde for 20 min to ensure that the retinal tissues were fixed completely. The fixed eyecups were washed with 1× PBS 3 times each at 5-min intervals and then passed through a series of sucrose solutions (S1888-5KG, Sigma Life Science, St. Louis, MO) from 15% to 30% (made in 1× PBS) for 1 h each or until the eye cup sank to the bottom of the tube. The eye cups were finally embedded in 1 part of Tissue Tek O.C.T. Compound (4583, Sakura Finetek, Torrance, CA) mixed with 20% sucrose solution mixture. The embedded eye cups were sectioned as 8-μm sections on cryostat (Leica CM3050S) and collected on clean Superfrost Plus slides (48311-703, VWR, Radnor, PA). Sections were stored at −80°C until use.

### Immunohistochemistry

The frozen retinal sections were taken out of −80°C and thawed at room temperature. The thawed retinal sections were rehydrated with 1× PBS and permeabilized with 0.1% Triton X-100 (0694-1L, VWR Life Science, Solon, OH) made in 10% normal donkey serum (NDS) (S30-100ML, Sigma) for 15 min. The permeabilized tissues was saturated with 10% NDS (made in 1× PBS) for 1 h at room temperature. The sections were probed with primary antibodies (see Table S1 for antibody details) diluted in 10% NDS overnight at 4°C. After the primary antibody incubation, the slides were washed with 1× PBS 3 times for 5-min intervals each at room temperature. Following the washes, the sections were incubated with Alexa Fluor-conjugated secondary antibodies (see Table S2 for antibody details) diluted in 10% NDS for 1 h in the dark at room temperature. The secondary antibodies were removed after 1 h and a few drops of 1 μg/mL DAPI (10236 276 001, Roche, Indianapolis, IN) made in 1× PBS were added to sections and incubated in the dark for 10 min at room temperature. Finally, the slides were washed with 1× PBS 3 times at 5-min intervals, and mounted with Fluoromount-G (17984-25, Electron Microscopy Sciences) and cover glasses (16004-350, VWR). Stained sections were imaged using LSM 700 inverted confocal microscope (Carl Zeiss, Thornwood, NY). Captured images were processed and montaged using ImageJ software (NIH).

## Supplemental information

Document S1. Figures S1–S4 and Tables S1 and S2

Document S2. Article plus supplemental information
