# Supplementary material for: Human iPSC-derived photoreceptor transplantation in the cone dominant 13-lined ground squirrel
Source: Stem Cell Reports. 2024 Feb 8;19(3):331–42. doi: 10.1016/j.stemcr.2024.01.005 (PMC10937153; doi:10.1016/j.stemcr.2024.01.005)
Supplement: Document S1. Figures S1–S4 and Tables S1 and S2 [file mmc1.pdf]

Stem Cell Reports, Volume *19*

Supplemental Information

Human iPSC-derived photoreceptor transplantation in the cone dominant 13-lined ground squirrel

Ching Tzu Yu, Sangeetha Kandoi, Ramesh Periasamy, L. Vinod K. Reddy, Hannah M. Follett, Phyllis Summerfelt, Cassandra Martinez, Chloe Guillaume, Owen Bowie, Thomas B. Connor, Daniel M. Lipinski, Kenneth P. Allen, Dana K. Merriman, Joseph Carroll, and Deepak A. Lamba

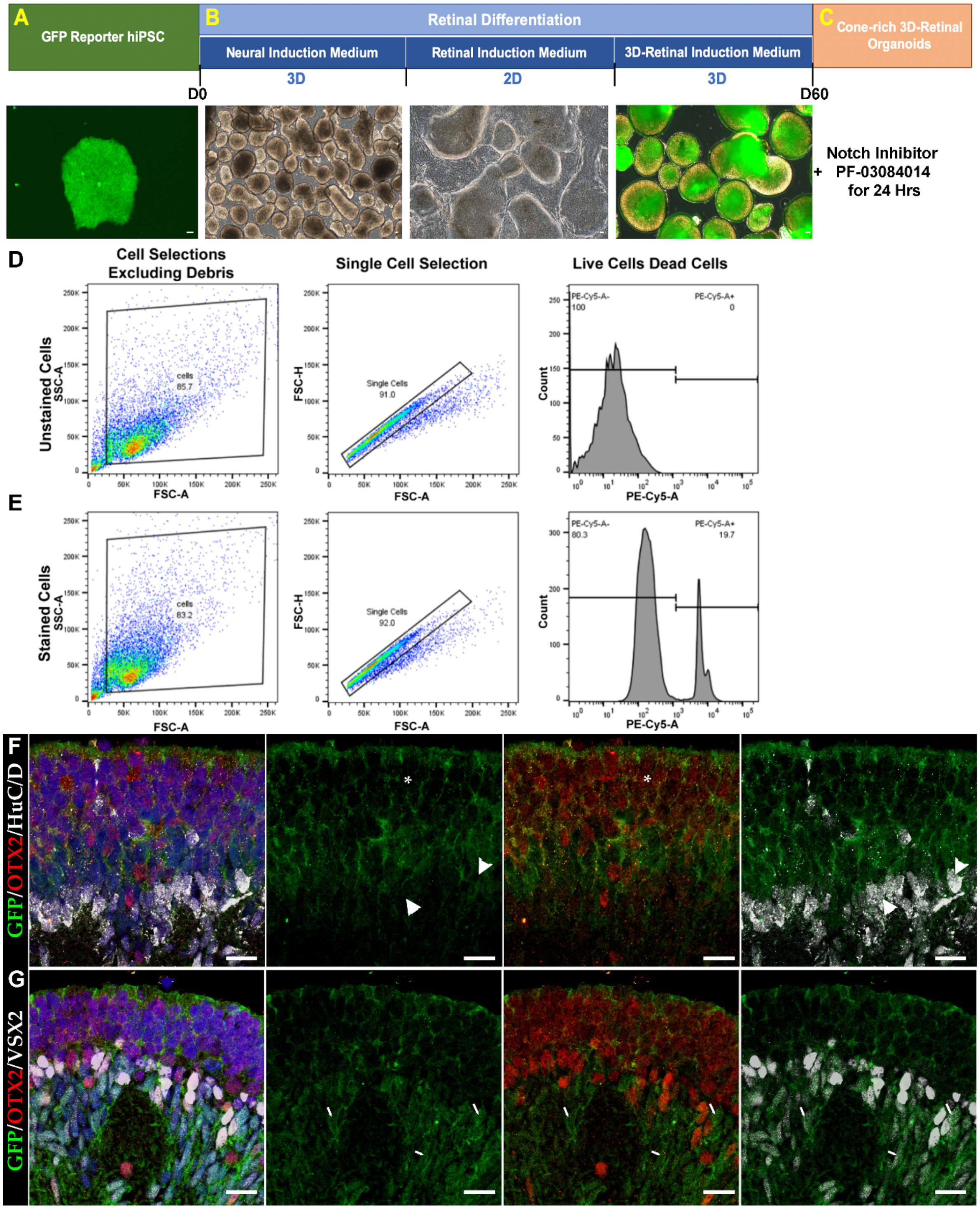

Figure S1. Cone-rich 3D-retinal organoids from hiPSC with pan-expressing GFP reporter (related to Figure 1). hiPSCs with GFP reporter knocked in at the safe harbor locus (A) were differentiated to generate the cone-rich 3D-retinal organoids using our previously described stepwise differentiation process (B) for ~8 weeks. 3D-retinal organoids were treated with a small molecule (PF) Notch pathway inhibitor for 24 hours (C). (D, E) Cell viability of single cells from dissociated organoids by flow cytometry. A representative plot of unstained cells (control) (D) and stained cells (E) acquisition showing the gated plot area of cells excluding debris, single cells selection excluding doublet populations, and a histogram plot showing two separate peaks of live and dead cells negative and positive for viability dye respectively. (F, G) Partial loss of GFP following organoid maturation (D150). Representative images showing loss of GFP in subsets of OTX2+ photoreceptors (highlighted by *) and HuC/D+ amacrine/ganglion cells (highlighted by arrowheads) in F but is well preserved in VSX2+ stem cells and bipolar cells (highlighted by arrowheads) in G. Scale Bar = 20μm.

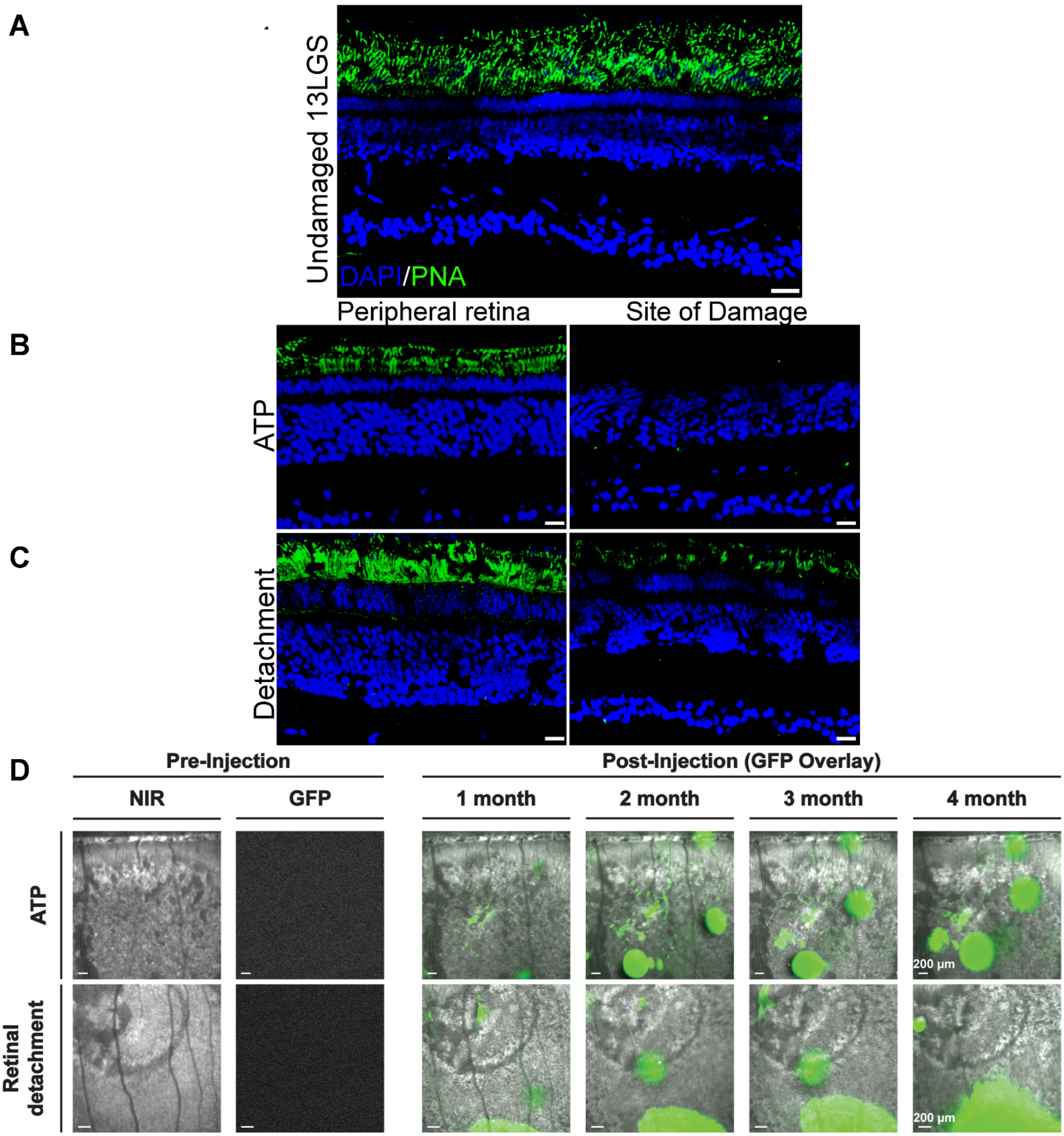

Figure S2 Assessment of cone loss and integration in 13LGS retinas following ATP injection or retinal detachment (related to Figure 1, 2). (A) Representative image showing PNA-FITC (green) marking normal cones outer segments in undamaged 13LGS and 2-3 cell deep photoreceptor layer upon DAPI (blue, nuclei) immunostaining. (B) Representative images of retina following ATP damage showing complete loss of photoreceptor nuclei and lack of any PNA-FITC at site of ATP injection while retina is relatively preserved in far periphery. (C) Representative images of retina following retinal detachment showing loss of photoreceptor nuclei (1-2 cell layers) and reduction in PNA-FITC while away from detachment site the retina is preserved. Scale Bar = 20μm. (D) Long-term survival of transplanted human iPSC-derived photoreceptors in 13-LGS retinas upon degeneration. NIR and fSLO images captured from the 13-LGS retinas with ATP, and retinal detachment induced damage models during pre- and post-transplantation. Longitudinal follow-up post-transplantation (fSLO images were overlayed on NIR) shows persistent GFP signals for up to 4 months.

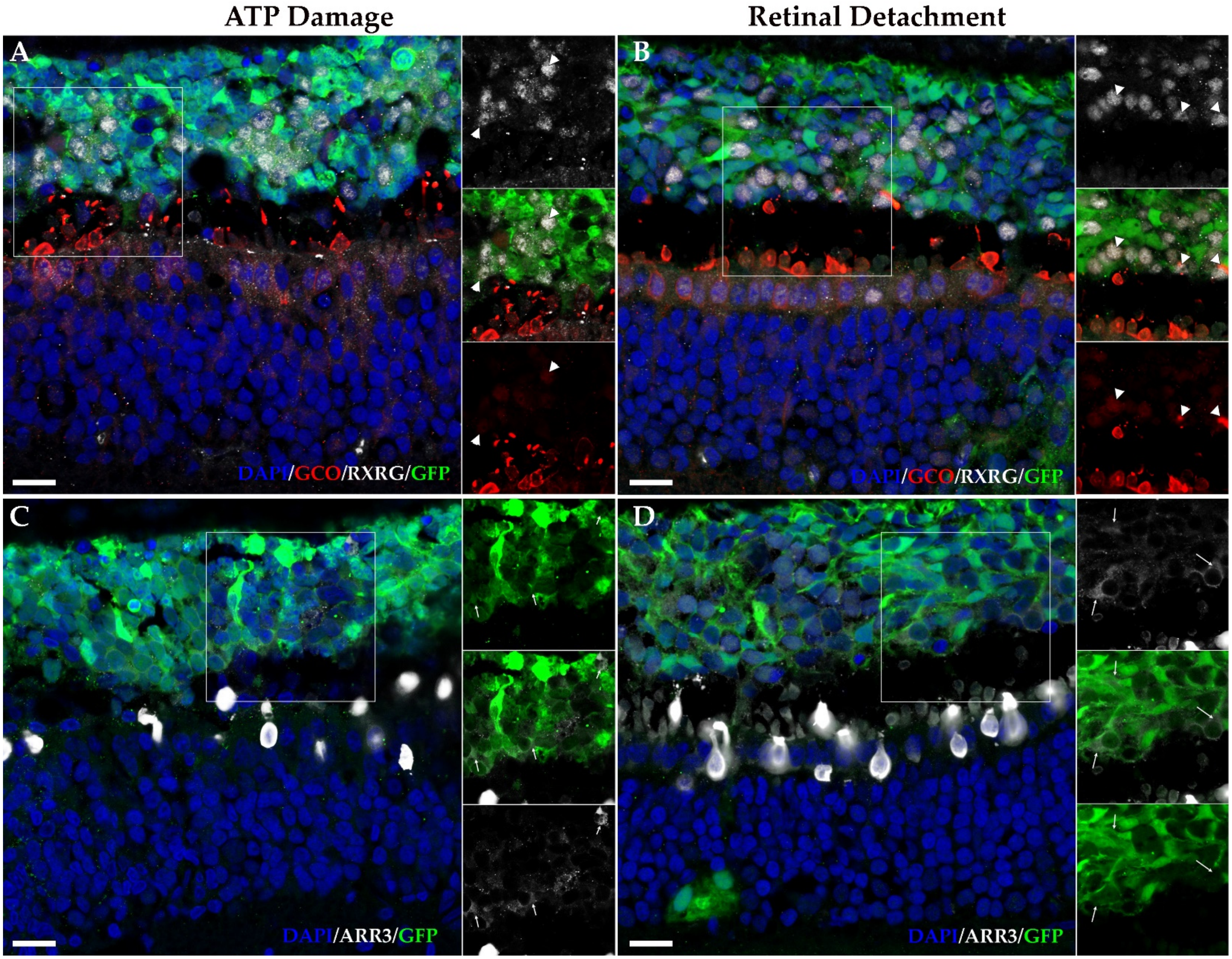

Figure S3 Expression of cone markers in the transplanted cells (related to Figures 3, 4). (A, B) Representative images showing expression of cone markers, green opsin (GCO, red) and RXRG (white) in GFP+ transplanted cells in ATP damage and retinal detachment eyes. Insets showing zoomed-in view and subpanels with co-expression highlighted by arrowheads. (C, D) Representative images showing expression of cone marker, ARR3 (white) in GFP+ transplanted cells in ATP damage and retinal detachment eyes. Insets showing zoomed-in view and subpanels with co-expression highlighted by arrows. DAPI (blue) marks nuclei. Scale Bar = 20μm.

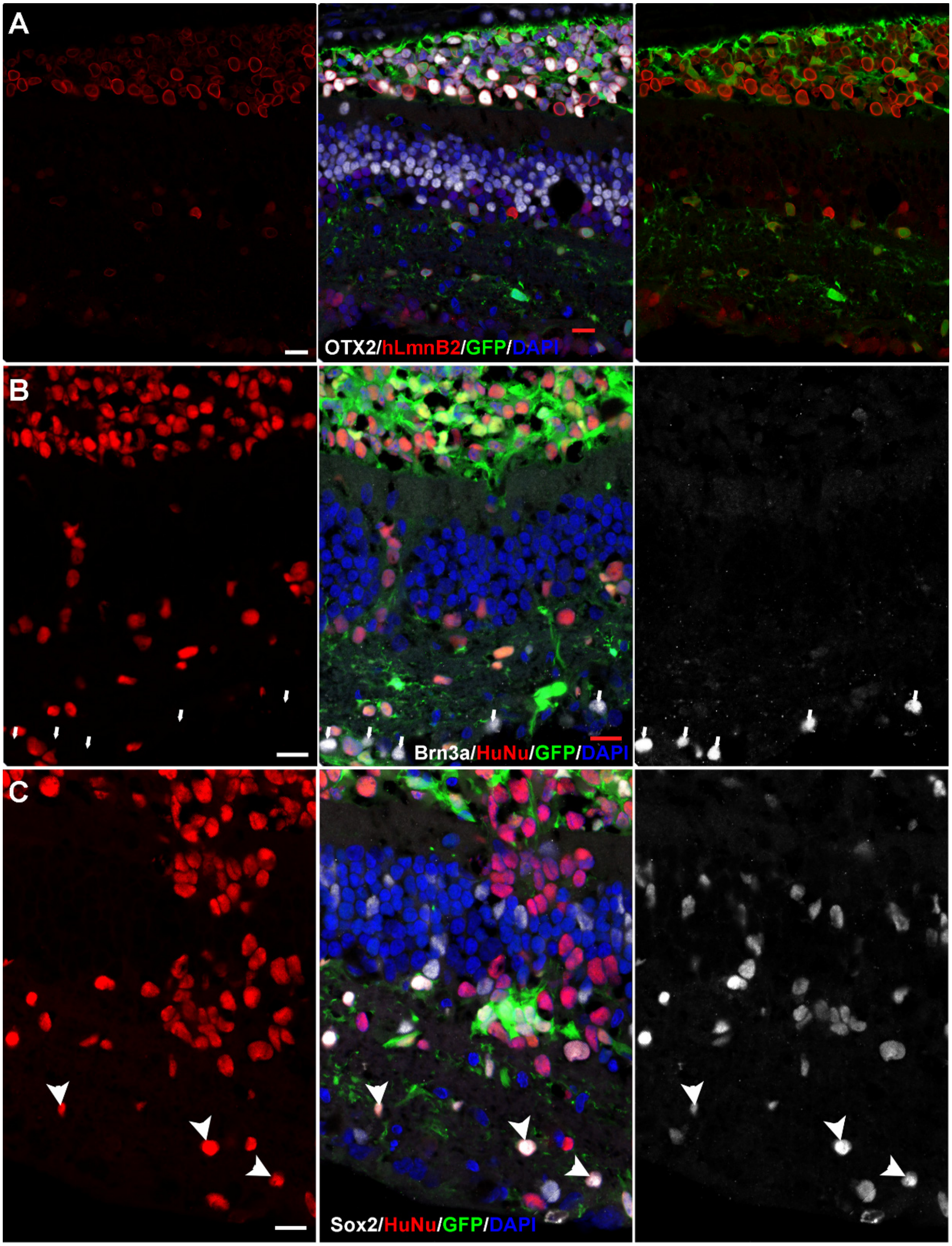

Figure S4 Expression of markers in the transplanted cells (related to Figures 3, 4). (A) Representative image showing expression of a human specific nuclear envelope marker, LMNB2 (red) in all GFP+ cells. Otx2(white) marks photoreceptor cells. (B) HuNu+ (red) and GFP+ human cells that migrated into the ganglion cell layer do not express Brn3A (white), a ganglion cell-specific marker (arrows). (C) Representative image showing that some of the HuNu+ (red) and GFP+ human cells that migrated into the inner retina co-express Sox2, a stem cell and glial marker. Arrowheads mark co-expression. DAPI (blue) marks nuclei. Scale Bar = 20µm.

Supplementary Table 1. Primary Antibody List **(related to Figures 3, 4)**.

Protein Name	Host	Dilution	Source	Cat. No.
Brn-3A	Mouse	1:100	Santa Cruz Biotechnology, Inc.	sc-8429
RXRG	Mouse	1:200	Santa Cruz Biotechnology, Inc.	sc-365252
GFP	Chicken	1:1000	Abcam	ab13970
GFP	Rabbit	1:500	GeneTex	GTX113617
HuC/D	Mouse	1:100	Life Technologies	A-21271
HuNu	Mouse	1:500	Millipore Sigma	MAB1281
Lamin B2	Mouse	1:500	GeneTex	GTX628803
Otx2	Goat	1:400	R&D Systems	BAF1979
Recoverin	Rabbit	1:1000	EMD Millipore	AB5585
GCO	Rabbit	1:500	EMD Millipore	AB5405
ARR3	Mouse	1:250	EMD Millipore	MABN2636
Sox2	Goat	1:400	R&D Systems	AF2018
Synaptophysin	Mouse	1:100	Invitrogen	14-6525-82
PNA-FITC	-	0.02 mg/ml	Thermo Scientific	L21409

Abbreviations: Brn-3A, brain-specific homeobox/POU domain protein 3A; GFP, green fluorescent protein; HuC/HuD, Hu-antigen D; HuNu, human nuclei; Otx2, Orthodenticle homolog 2; GCO, Green Cone Opsin; ARR3, cone arrestin; RXRG, retinoid X receptor gamma; Sox2, SRY-box 2.

Supplementary Table 2. **(related to Figures 3, 4)**.

Species	Target	Conjugate	Dilution	Source	Cat. No.
Donkey	Anti-mouse	Alexa Fluor 488	1:250	Invitrogen	A-21202
Donkey	Anti-mouse	Alexa Fluor 555	1:250	Invitrogen	A-31570
Donkey	Anti-mouse	Alexa Fluor 647	1:250	Invitrogen	A-31571
Donkey	Anti-rabbit	Alexa Fluor 488	1:250	Invitrogen	A-21206
Donkey	Anti-rabbit	Alexa Fluor 555	1:250	Invitrogen	A-31572
Donkey	Anti-rabbit	Alexa Fluor 647	1:250	Invitrogen	A-31573
Donkey	Anti-Goat	Alexa Fluor 488	1:250	Invitrogen	A-11055
Donkey	Anti-Goat	Alexa Fluor 555	1:250	Invitrogen	A-21432
Donkey	Anti-Goat	Alexa Fluor 647	1:250	Invitrogen	A-21447
Goat	Anti-mouse	Alexa Fluor 555	1:250	Invitrogen	A-21127
Goat	Anti-mouse	Alexa Fluor 647	1:250	Invitrogen	A-21242
